# Suppressive Oligodeoxynucleotides Promote the Development of Th17 Cells

**DOI:** 10.1371/journal.pone.0067991

**Published:** 2013-07-02

**Authors:** Christian Bode, Xiang-Ping Yang, Hiu Kiu, Dennis M. Klinman

**Affiliations:** 1 Cancer and Inflammation Program, National Cancer Institute, National Institutes of Health (NIH), Frederick, Maryland, United States of America; 2 Molecular Immunology and Inflammation Branch, National Institute of Arthritis, Musculoskeletal and Skin Diseases, National Institutes of Health (NIH), Bethesda, Maryland, United States of America; Charité, Campus Benjamin Franklin, Germany

## Abstract

Synthetic oligonucleotides containing repetitive TTAGGG motifs mimic the immunosuppressive activity of telomeric DNA. These suppressive oligonucleotides (Sup ODN) are effective in the treatment/prevention of various inflammatory and autoimmune diseases in mice. The therapeutic activity of Sup ODN was originally attributed to the inhibition of Th1 cell activation. Current results indicate that Sup ODN also promote the maturation of naive CD4^+^ T cells into Th17 effectors. The generation of Th17 cells is linked to the prolonged activation of signal transducer and activator of transcription (STAT)3 mediated by suppressor of cytokine signaling 3 (SOCS3) inhibition. *In vivo* studies show that treatment with Sup ODN promotes Th17 responsiveness under physiological conditions, increasing host resistance to *Candida albicans* infection. These findings support the development of Sup ODN to suppress pathological inflammatory conditions and improve host resistance to fungal pathogens.

## Introduction

DNA has multiple and complex effects on the immune system. For example, the repetitive TTAGGG motifs present in the telomeric ends of mammalian chromosomes have immunosuppressive properties [1]. Synthetic oligonucleotides (ODN) containing repetitive TTAGGG motifs mimic the ability of telomeric DNA to prevent and treat a variety of inflammatory and autoimmune diseases [2]–[7] This suppressive activity was initially attributed to the ability of Sup ODN to inhibit the differentiation of naive CD4^+^ T cells into Th1 effectors by blocking the phosphorylation of STAT1 and STAT4, thereby reducing the production of IFN_γ_ which is critical for the maintenance of the Th1 response [5], [8], [9]. These changes indirectly supported the generation of Th2-dominated immune responses. That mechanism was proposed when the Th1:Th2 ratio was considered the major determinant of host susceptibility to autoimmune disease, before the discovery of Th17 cells.

Th17 cells and the effector molecules they produce (including IL-17A and IL-17F) are now known to contribute to the pathogenesis of inflammatory and autoimmune diseases [10]. In this context, elevated levels of IL-23 support the expansion of autoreactive Th17 cells, indicating that Th17 cell pathogenicity is influenced by the cytokine milieu in which they arise [11]–[16]. Th17 cells also play a critical role in defending the host from extracellular pathogens, as widely demonstrated by studies involving infection with *Candida albicans* [17].

Th17 cells are generated from naive precursors under the influence of IL-6 and TGF_β_ [18], [19]. IL-6 initiates Th17 differentiation by activating STAT3. TGF_β_ prolongs STAT3 activation by inhibiting the expression of SOCS3 (suppressor of cytokine signaling 3), a major negative regulator of STAT3-activating cytokines [20]–[23]. The development of Th17 cells is strongly inhibited by the Th1 cytokine IFN_γ_ and the Th2 cytokine IL-4 [24].

Reports published over a decade ago established that Sup ODN prevented or reduced the severity of autoimmune diseases including lupus and collagen-induced arthritis in murine models [2], [3]. Current understanding of the role of Th17 cells in the development of these diseases [15], [25], [26] raises intriguing possibility that Th17 cells may be targeted by Sup ODN. The current studies are the first to explore the ability of Sup ODN to modulate the activation and differentiation of Th17 cells. Contrary to expectations, we find that Sup ODN promote the maturation of naive precursors into Th17 effectors. This was observed both *in vitro* and under physiological conditions *in vivo*, where the resultant Th17 cells increased host resistance to *Candida* challenge. The mechanism underlying this activity reflects the ability of Sup ODN to inhibit the expression of SOCS3, leading to prolonged activation of STAT3 and the persistent generation of Th17 cells.

## Materials and Methods

### Mice and Media

Female C57BL/6 mice were bred at the National Cancer Institute (Frederick, MD) and studied at 6–10 wk of age. All animal studies were performed according to National Institutes of Health guidelines for the use and care of live mice and were approved by the Institutional Animal Care and Use Committee of the National Cancer Institute at Frederick (ASP 12–459).

Cells were maintained in RPMI 1640 medium supplemented with 10% FCS (both from Lonza, Walkersville, MD), 2 mM glutamine, 100 IU/ml penicillin, 100 µg/ml streptomycin and 25 mM HEPES buffer (all from Invitrogen, Carlsbad, CA), and 0.0035% 2-ME (Sigma-Aldrich, St. Louis, MO).

### Oligodeoxynucleotides

Phosphorothioate ODN were synthesized at the Core Facility of the Center for Biologics Evaluation and Research facility, Food and Drug Administration (Bethesda, MD). The following ODN were used: suppressive ODN A151 (5′-TTAGGGTTAGGGTTAGGGTTAGGG-3′) and control ODN 1612 (5′-GCTAGAGCTTAGGCT-3′). All ODN were free of detectable protein or endotoxin contamination.

### T Cell Isolation and Differentiation

Naive CD4^+^ T cells were purified from C57BL/6 splenocytes by negative selection and magnetic separation (Miltenyi Biotec, Auburn, CA) followed by sorting of the naïve CD4^+^/CD62L^+^/CD44^−/^CD25^−^ population using a FACSAria II (BD Biosciences, Jan Jose, CA). PerCP–Cy5.5–anti-CD4 (RM4-5), FITC–anti-CD62L (MEL-14), PE–anti-CD44 (IM-7) and APC-anti-CD25 (PC61) were used for cell sorting (all reagents from BD Biosciences). Naïve CD4^+^ T cells (0.5×10^6^/ml) were incubated with or without 1 µM ODN for 2 h and then activated for 3–5 d by 3 µg/ml plate-bound anti-CD3 (2C11; BD Biosciences) plus 2 µg/ml plate-bound anti-CD28 (37.51; BD Biosciences).

Cells were stimulated under neutral conditions or Th17-biased conditions, the latter involving the addition of 20 ng/ml IL-6 (R&D Systems, Minneapolis, MN) plus 0.1 ng/ml human TGF-β1 (R&D Systems), 10 µg/ml anti-IFNγ (XMG1.2), 10 µg/ml anti-IL-4 (11B11) and 10 µg/ml anti–IL-2 (S4B6; all from BD Biosciences). After differentiation, cells were stimulated for 4 h with 50 ng/ml PMA and 500 ng/ml ionomycin (both from Sigma-Aldrich) in the presence of 1 mg/ml brefeldin A (GolgiPlug; BD Biosciences). The cells were fixed in 4% formyl saline, permeabilized with 0.1% saponin buffer, and stained with fluorescence-linked Abs then evaluated using a LSRFortessa cell analyzer (BD Biosciences). Events were collected and analyzed with FlowJo software (Tree Star, Ashland, OR). The following Abs were used: PE-anti-IL-17A (18H10), APC-anti-IFNy (XMG1.2), PE-Cy7-anti-IL-4 (11B11; all from BD Biosciences). Cell culture supernatants were assayed for IL-17A and IL-17F by using Duo-Set ELISA (R&D Systems) as recommended by the manufacturer.

### *C. albicans* and Culture Conditions

*C. albicans* (strain CAM15.4) was obtained from Dr. Malcom Whiteway (National Research Council of Canada, Montreal, Canada) and maintained on 1% yeast extract, 2% peptone and 2% dextrose agar (YPD agar; BD Difco, Sparks, MD). For the preparation of *C. albicans* blastoconidia inocula, a yeast colony was transferred to 250 ml YPD broth (BD Difco) and incubated for 18 h in a 30°C incubator shaker rotating at 150 r.p.m., Cells were visually characterized and then enumerated by use of a haemocytometer. Aliquots of blastospores were frozen in sterile PBS supplemented with 50% glycerol at −70°C and thawed in pyrogen-free PBS immediately prior to use. The number of viable *C. albicans* cells was determined by plating serial dilutions on YPD agar.

### *C. albicans* Infection Model

Female C57BL/6 mice were infected via the tail vein with 10^5^ CFU of *C. albicans* blastoconidia in 100 µl of sterile pyrogen-free PBS. Half of the mice were treated by i.p. injection of 300 µg of Sup ODN 3 h before and 3 d after challenge. Body weight was measured at baseline and then daily until the animals were sacrificed on day 5. The severity of infection was determined by culturing serial 10-fold dilutions of homogenized kidney preparations in YPD agar. The remaining kidney tissue was centrifuged and the supernatant was used to measure IL-17A and IL-17F levels as previously described by using Duo-Set ELISA (R&D Systems) [27]. To assess the effect of Sup ODN on Ag-specific Th17 differentiation *in vivo*, splenocytes were harvested on day 5 and then cultured at 2×10^6^ cells/well in a 96-well U-bottomed plate at 37°C in 5% CO_2_ for 2 days with 10^5^ heat-killed (70°C for 45 min) *C. albicans*. Duo-Set ELISA (R&D Systems) was used to measure the IL-17A levels in the supernatant.

### RNA Isolation and Quantitative Real-time PCR

Total RNA was isolated from cultured T cells using the RNeasy Mini Kit (Quiagen, Valencia, CA); cDNA was synthesized with a QuantiTect Reverse Transcription kit according to the manufacturer’s instructions (Applied Biosystems, Carlsbad, CA). Gene expression levels (normalized to GAPDH) were analyzed using the StepOnePlus RT-PCR system (Applied Biosystems). All reagents and probes used in these studies were purchased from Applied Biosystems. The following TaqMan assays were used: RORC (Mm01261022_m1), T-bet (Mm00450960_m1), IL-23R (Mm00519943_m1), IL-33 (Mm00505403_m1), IL-10 (Mm00439616_m1) and SOCS3 (Mm00545913_s1).

### Flow Cytometric Analysis of Phospho-Stat3 Expression

Naive T cells were cultured under Th17 polarizing conditions±Sup ODN. Cells were fixed with BD Lyse/Fix Buffer for 10 min at 37°C, washed, permeabilized in ice-cold BD Perm Buffer III for 30 min and then stained with PE-anti-Stat3 (pY705) Ab (all reagents from BD Biosciences) for 30 min at RT. Flow cytometric analysis was performed on a LSRFortessa cell analyzer. The percent reduction in pSTAT3 levels was calculated by the formula:

(1 - (level of STAT3 phosphorylation at 20 h - pSTAT3 plateau level) ) X 100% (peak pSTAT3 level - pSTAT3 plateau level).

### Statistical Analysis

Statistical analyses was performed using GraphPad Prism 5 (GraphPad Software, La Jolla, CA). Student’s t test was used to examine differences in cell populations, cytokine levels, and fungal growth between groups. Weight loss was analyzed by two-way ANOVA. Values of p<0.05 were considered significant. All values are expressed as means ± SE unless otherwise noted.

## Results

### Suppressive ODN Promote the Generation of Th17 Cells *in vitro*

To examine whether Sup ODN influenced the maturation of Th17 cells, culture conditions were identified that generated large but not optimal numbers of Th17 cells *in vitro*. This strategy would allow us to detect either an increase or decrease in Th17 frequency after Sup ODN treatment. The conditions selected used conventional amounts of the Th17 polarizing cytokines TGF_β_ and IL-6 but lower concentrations of anti-CD3 and anti-CD28 Abs to stimulate T cell expansion. As Sup ODN reduce IFN_γ_ production but increase IL-4 levels under Th1 polarizing conditions [8], both anti-IFN_γ_ and anti-IL-4 Abs were included during culture to limit any indirect influence of Sup ODN on Th17 cell generation.

**Figure 1:**
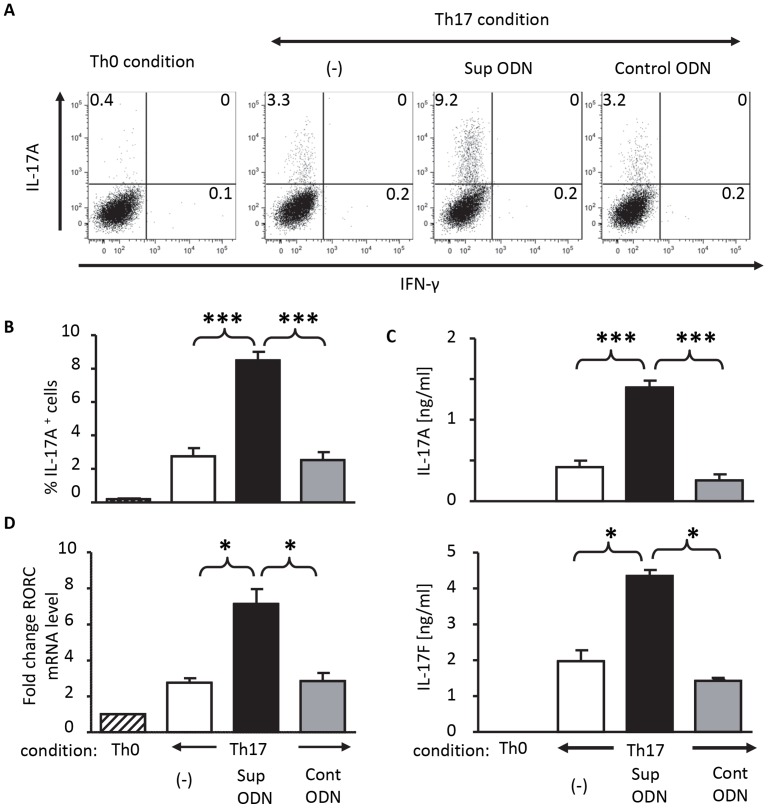
Suppressive ODN promote the maturation of Th17 cells. Highly purified naive CD4^+^ T cells from female C57BL/6 mice were incubated for 2 h with 1 µM of suppressive or control ODN and then cultured for 3 days under Th0 or Th17 polarizing conditions (see methods section for details). (A) Frequency of cells producing IL-17A and IFN_γ_ in samples as determined by intracellular staining and flow cytometry. (B) Combined results (from 4 independent experiments) showing the mean+SD of the effects of treatment on the frequency of IL-17A^+^ cells. (C) Level of IL17A and IL-17F protein in culture supernatants of cells stimulated under Th17 polarizing conditions for 3 days (mean+SD of 4 experiments). (D) Level of mRNA encoding RORC determined by RT-PCR. mRNA levels in cells treated under Th17 polarizing conditions are compared to those generated under Th0 conditions after normalization to GAPDH mRNA for each sample. Each bar represent the mean+SD of 3 independent experiments. *, p<0.05; ***, p<0.001.

The inclusion of Sup ODN increased the number of Th17 cells generated from highly purified naive CD4^+^ T cells by ≈3-fold when compared to control cultures (Fig. 1 A,B; p<0.001). This effect was sequence specific, as control ODN lacking suppressive TTAGGG motifs did not influence the generation of Th17 cells (Fig. 1 A,B). Previous studies established that Sup ODN could inhibit the generation of Th1 cells thereby increasing Th2 responses [5], [8], [9]. To evaluate whether the effect of Sup ODN on Th17 cell differentiation might be influenced by changes in the Th1:Th2 balance, the maturation of such cells was evaluated. Under the Th17 polarizing conditions used in the current work (which included Abs that blocked Th1 and Th2 cell differentiation) the frequency of IFN_γ_ and IL-4 secreting cells was very low. This frequency was not altered by the presence of Sup ODN (Fig 1A and Fig. S1).

To determine whether the Th17 cells present *in vitro* were biologically active, their ability to produce IL-17A and IL-17F was examined. As seen in Fig. 1C, these Th17 cytokines were significantly elevated in the culture supernatants of cells treated with Sup ODN when compared to controls (IL-17A; p<0.05; IL-17F; p<0.05). These supernatants did not contain detectable levels of IL-21 or IL-22 (data not shown). As Th17 development relies on the lineage-specific transcription factor orphan nuclear receptor ROR-γt (RORC) [28], expression of that gene was monitored under Th17 polarizing conditions. Cells cultured in the presence of Sup ODN had significantly elevated RORC mRNA levels compared to controls, consistent with the increased numbers of Th17 cells present in Sup ODN treated cultures (Fig 1D; p<0.05). This series of findings suggests that Sup ODN directly support the generation of Th17 cells. Suppressive ODN increase the Th17 response and reduce fungal burden in *C. albicans* infected mice.

To examine the effect of Sup ODN on Th17 cells under physiologically relevant conditions, a well established murine model of *Candida* infection was employed. Th17 cells play a critical role in protecting the host from *C. albicans* infection (17) in that elevated IL-17 levels are associated with lower fungal burdens and improved survival [27]. Previous studies using this model showed that systemic fungal challenge induced a significant increase in the number of Th17 cells present *in vivo* [26], [29]. This effect peaked 5 days after challenge and was best assessed in the kidneys, the site where pathogen burden is highest [27], [30], [31].

**Figure 2:**
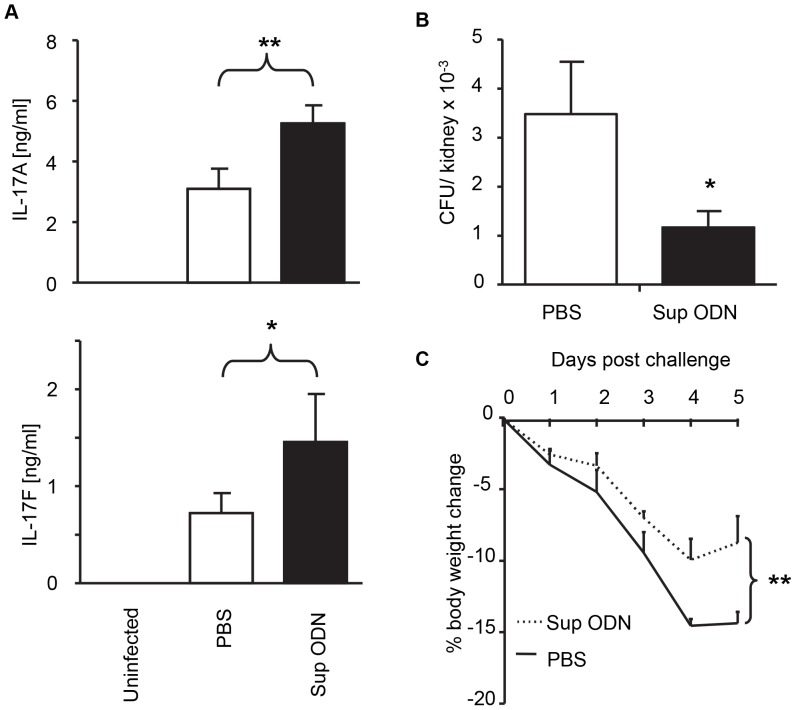
Effect of Suppressive ODN on *C.* *albicans* infection of mice. C57BL/6 mice were challenged i.v. with 10^5^ CFU of *C. albicans.* 3 h before and 3 days after challenge these mice were treated i.p. with 300 µg of Sup ODN or PBS. On day 5, the kidneys were removed, homogenized and centrifuged. (A) The supernatant from each kidney was analyzed for the presence of IL-17A and IL-17F while (B) the homogenate was plated to quantify viable *Candida*. Weight loss was also monitored (C). Data in are representative of three independent experiments involving a total of 15 mice/group. *, *p*<0.05. **, p<0.01 vs PBS.

**Figure 3:**
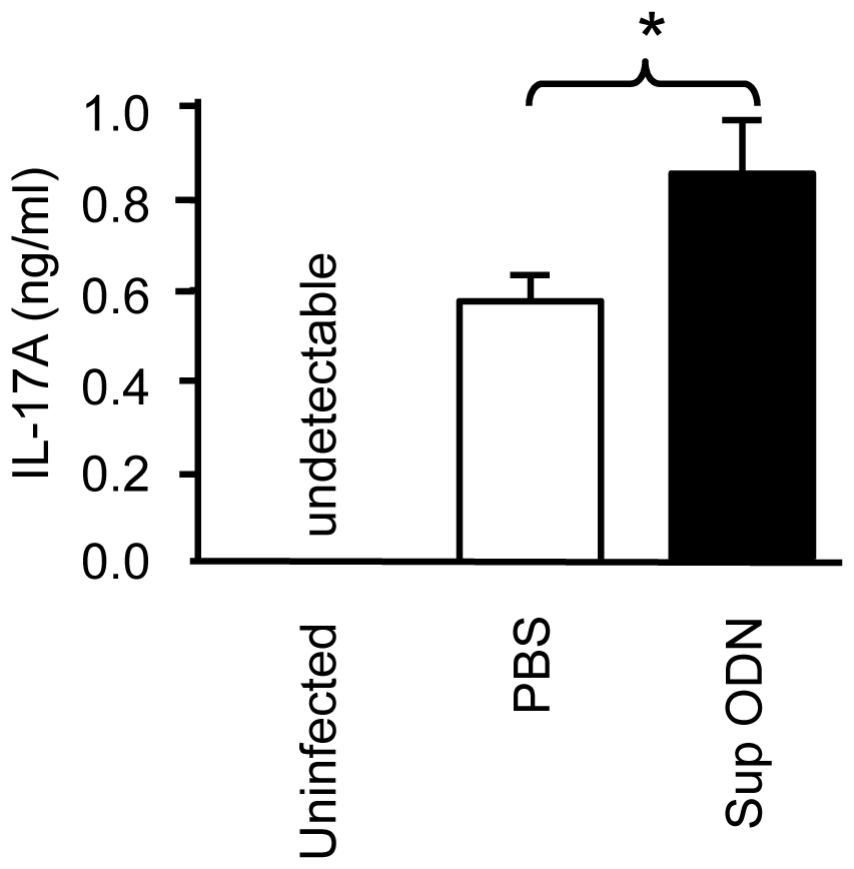
Suppressive ODN improve the Ag-specific Th17 response induced by ***C.*** *albicans.* C57BL/6 mice were challenged with *C. albicans* and treated with Sup ODN or PBS as described in Fig 2. On day 5, single cell suspensions were prepared from the spleens of these mice and stimulated *ex vivo* with heat-killed *C. albicans* for 2 d. Culture supernatants were analyzed for IL-17A levels by ELISA. Data represent the mean+SE of two experiments involving 4–5 independently studied animals/group. Note that no cytokine was detected in unstimulated cultures or stimulated cultures from uninfected mice. *, *p*<0.05 vs PBS.

Consistent with the literature, neither IL-17A nor IL-17F could be detected in the kidneys of normal uninfected mice (Fig. 2A) [27]. Five days post i.v. challenge with 10^5^ vegetative *C. albicans*, both cytokines were present in all kidneys (Fig. 2A). Challenged mice treated with Sup ODN had even higher levels of these cytokines (p<0.05, Fig 2A). To evaluate the specificity of this response, spleen cells from infected mice were harvested on day 5 and stimulated *ex vivo* with heat-killed *C. albicans*. As seen in Fig. 3, splenocytes from the Sup ODN treated donors produced significantly more IL-17A than did those from infection-matched controls (p<0.05). However Sup ODN treatment had no effect on IFN_γ_ levels (data not shown), consistent with the *in vitro* findings noted above.

Whether the increased Th17 response generated by Sup ODN treatment improved resistance to fungal infection was then evaluated. Mice were challenged with *Candida* and kidney fungal burden evaluated 5 days later. Consistent with previous reports showing that host resistance to infection correlates with increased IL-17 levels [27], [31], animals treated with Sup ODN had significantly lower numbers of infectious *Candida* in their kidneys than did PBS treated controls (Fig. 2B; p<0.05). Analyzing the weight of these animals provided additional evidence that Sup ODN treatment improved outcome. Weight loss is an accepted surrogate marker of the severity of fungal infection [32], [33]. As expected, body weight fell by nearly 15% in mice challenged with *C. albicans.* The magnitude of this weight loss was decreased by >40% when infected animals were treated with Sup ODN (Fig. 2C; p<0.01).

### Profile of Th17 Cells Generated by Sup ODN

**Figure 4:**
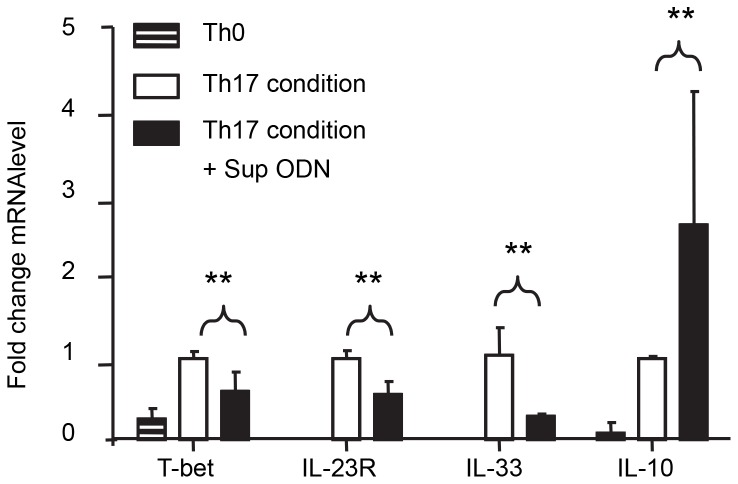
Gene expression by Th17 cells. Naive T cells from C57BL/6 mice were cultured under Th17 polarizing conditions as described in Fig 1. mRNA was isolated on day 3 and the expression of genes encoding T-bet, IL-23R, IL-33 and IL-10 determined by RT-PCR. Relative mRNA levels were calculated by comparison to Th17 cells generated in the absence of ODN after normalization to GAPDH mRNA levels. Each bar represents the mean+SD of 3 independent experiments. **, p<0.01.

Th17 cells are functionally heterogeneous: some contribute to the development of autoimmune disease while others protect the host from infection [34], [35]. These subpopulations are phenotypically distinct: Th17 cells that mediate autoimmunity are characterized by high levels of IL-23R, IL-33 and T-bet expression when compared to their non-pathogenic counterparts that express elevated levels of IL-10 [11], [12]. To clarify the nature of the cells generated by Sup ODN treatment, mRNA levels of those genes known to distinguish between autoimmune vs protective Th17 cells were examined. As seen in Fig. 4, Th17 cells generated in cultures supplemented with Sup ODN expressed significantly lower levels of mRNA encoding T-bet (≈2-fold, p<0.01), IL-23R (2-fold, p<0.01) and IL-33 (3.5-fold, p<0.01) while expressing elevated levels of IL-10 mRNA (≈2.5-fold, p<0.01) when compared to cells raised in the absence of Sup ODN.

### Sup ODN Promote the Generation of Th17 Cells by Inhibiting SOCS3 Expression, Thereby Prolonging STAT3 Activation

Previous work established that IL-6 and TGF_β_ support the generation of Th17 cells via a STAT3-dependent mechanism [20], [36], [37]. In contrast, the presence of IFN_γ_, IL-4, IL-27 and/or IL-2 inhibit the maturation of Th17 cells via mechanisms involving STAT1 and STAT5 [24], [38], [39]. When culture conditions favoring the development of Th1 cells are employed, Sup ODN alter the Th1:Th2 ratio by influencing STAT1 expression [8]. Thus, the possibility that Sup ODN altered Th17 cell maturation via a STAT dependent mechanism was investigated.

**Figure 5:**
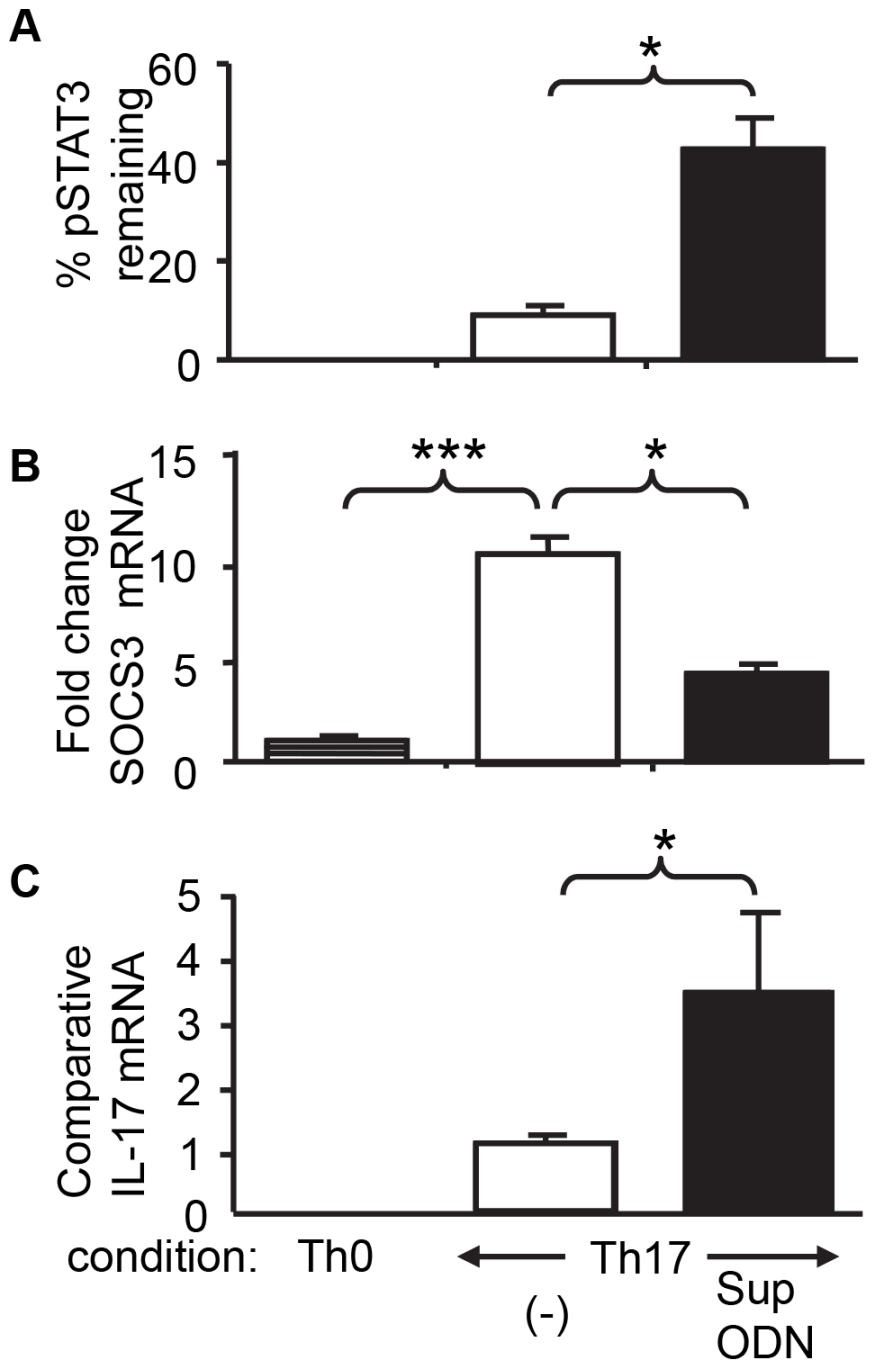
Effect of Suppressive ODN on STAT3, SOCS3 and IL-17. Naive T cells from C57BL/6 mice were cultured under Th17 polarizing conditions for 20 h as described in Fig 1. A) The level of STAT3 phosphorylation was determined by flow cytometry using a phospho-specific anti-STAT3 Ab. Results reflect the increase over baseline in phosphorylated STAT3 vs peak levels (see methods section for details). B,C) mRNA was isolated and analyzed by RT-PCR. The level of SOCS3 and IL-17 expression was normalized to GAPDH levels in the same sample. Changes in SOCS3 expression determined by comparison to cells cultured under Th0 biased conditions. Data represent the mean+SD of 3 independent experiments. *, p<0.05; ***, p<0.001.

Highly purified naive T cells were cultured under conditions that favored the generation of Th17 cells (using medium supplemented with IL-6 and TGF_β_). The phosphorylation of STATs 1, 3 and 5 was evaluated over a 72 h period. Preliminary studies showed that STAT3 was not phosphorylated in resting cells (0.9±0.2%). Upon stimulation, tyrosine phosphorylation rose rapidly to peak at 0.5 h (96.2±2.4%) followed by a slow decline over the next 48 h to a plateau level that persisted through 72 h (56.4±0.8%). The addition of Sup ODN did not increase the already high level of STAT3 phosphorylation at 0.5 h (96.1±2.1%) but did slow the rate at which STAT3 dephosphorylated. At 20 h (when IL-17 gene expression first became detectable) the level of STAT3 phosphorylation had fallen to 59.6±1.7% in control cultures (barely above plateau levels) while in Sup ODN treated cells STAT3 phosphorylation was at 73.7±2.5% (>40% above plateau values)(Fig. 5 A, p. <0.05). These findings suggest that Sup ODN prolonged the duration of STAT3 phosphorylation during the process of Th17 cell differentiation. By comparison, Sup ODN had no effect on levels of STAT1 or STAT5 phosphorylation under Th17 polarizing conditions at any time point (Figure S2).

Previous studies showed that SOCS3 acts as a negative regulator of STAT3 phosphorylation in Th cells, and that inhibiting SOCS3 prolongs STAT3 activation during Th17 cell differentiation [21], [22]. To determine whether the prolonged STAT3 activation observed when cells were treated with Sup ODN involved the same mechanism, the expression of SOCS3 mRNA was monitored. As predicted, Sup ODN reduced SOCS3 mRNA levels by 2-fold compared to control cells, an effect that was detectable by 0.5 h and persisted for 48 h (Fig. 5B and data not shown; p<0.05). These results suggest that Sup ODN promotes the generation of Th17 cells by inhibiting SOCS3, thereby prolonging the phosphorylation of STAT3.

## Discussion

This study is the first to examine the effect of Sup ODN on the generation of Th17 cells. Results demonstrate that Sup ODN promote the differentiation of naive CD4^+^ T cells into Th17 effectors *in vitro* (Fig. 1) and *in vivo* (Figs. 2,3). As a result, Sup ODN treatment improves host resistance to *Candida* infection, as a robust Th17 response promotes the elimination of fungal pathogens. Mechanistically, Sup ODN down-regulated SOCS3 expression, allowing for the prolonged activation of STAT3 in support of Th17 cell maturation (Fig. 5). The profile of genes expressed by cells generated by Sup ODN classifies them among the Th17 subpopulation that does not mediate autoimmunity (Fig. 4). Consistent with that finding, multiple studies showed that Sup ODN treatment protects mice against inflammatory and autoimmune diseases [2]–[4].

Sup ODN are composed of repetitive TTAGGG motifs identical to those found at high frequency in mammalian telomeres. Sup ODN replicate the ability of telomeric DNA to reduce inflammation and autoimmunity [2], [3], [6], [7], [9]. Early studies found that Sup ODN could modulate the balance between Th1 and Th2 cells by inhibiting the differentiation of naïve CD4^+^ cells into Th1 effectors and postulated that this explained their therapeutic utility [5], [8]. Yet those reports predated the discovery of Th17 cells now known to have an important role in autoimmunity, inflammation, and the host response to extracellular pathogens [40]. Current findings using Th17 polarizing conditions show that Sup ODN directly promote Th17 cell differentiation without altering the frequency of Th1 or Th2 cells (Fig. 1 and S1). This effect is sequence specific, as control ODN had no effect on either the generation or activity of Th17 cells (Fig. 1).

The conditions used to generate Th17 cells result in the rapid phosphorylation of STAT3 [20], [36]. STAT3 phosphorylation is negatively regulated by SOCS3. High levels of SOCS3 inhibit STAT3 activation and reduce the generation of Th17 cells while the absence of SOCS3 enhances STAT3 phosphorylation and Th17 development [21], [23]. Current results show that Sup ODN inhibits the expression of SOCS3, thereby maintaining STAT3 activation and increasing the generation of Th17 cells (Fig. 5). Consistent with this finding, Qin et al demonstrated that TGF_β_ mediated inhibition of SOCS3 prolongs STAT3 activation and may promote Th17 differentiation [22].

Other members of the STAT family can also inhibit Th17 cell development [24], [38], [39]. Although Sup ODN were found to inhibit STAT1 activation in Th1 cells [8], we observed no effect of Sup ODN on STAT1 or STAT5 phosphorylation under Th17 polarizing conditions (Figure S2). This presumably reflects differences in the conditions that induce Th0 cells to differentiate into Th1 vs Th17 cells.

Th17 cells play an important role in protecting the host from extracellular pathogens [41]. This is exemplified by their ability to improve host resistance against *C. albicans* infection, the most common human fungal pathogen and a major cause of opportunistic infections in immunosuppressed individuals [17]. Evaluating the anti-fungal activity of the Th17 cells generated by Sup ODN treatment in a murine model of systemic candidiasis provided a physiologically relevant way of examining their function [29], [42]. Administering Sup ODN to pathogen-challenged mice improved Ag-specific Th17 cell immunity. Spleen cells from these animals produced significantly more IL-17 than those from infected controls when exposed to heat-killed Candida *ex vivo* (Fig. 3). The burden of Candida in the kidneys of infected animals (the dominant site of pathogen infection) was significantly reduced by Sup ODN treatment and IL-17 levels in the kidneys were significantly increased (Fig. 2). These findings are consistent with work documenting that elevated levels of IL-17 reduce host susceptibility to and mortality from *C. albicans* [27]. While we did not evaluate the effect of Sup ODN on survival (due to restrictions imposed by our ACUC), Sup ODN treatment did reduce weight loss in challenged mice and weight loss is a validated predictor of survival after systemic *Candida* challenge (Fig. 2C) [32], [33]. This constellation of findings supports the conclusion that the Th17 cells generated by Sup ODN *in vivo* promote host immunity against fungal infections.

Th17 cells are not the sole source of IL-17. Elements of the innate immune system including γδ T cells, lymphoid-tissue inducer-like cells and invariant natural killer T cells also secrete that cytokine [43]–[45]. Yet it is unlikely that the IL-17 response observed five days after Sup ODN treatment derived from these immune cells. The contribution of the innate immune system to IL-17 production peaks within 24 h of systemic Candida challenge and lasts for only 48 h [43], [44]. In contrast, the adaptive response involving Ag-specific Th17 cells matures over a period of 3–5 days [44]. By analyzing the effect of *C. albicans* at the peak of infection on day five, this study focused on the adaptive response of Th17 cells rather than elements of the innate immune system.

While some Th17 cells play a beneficial role by protecting the host from extracellular pathogens others cause harm by increasing host susceptibility to autoimmune disease. The environment in which a Th17 cell arises influences its effector function [34], [35]. A milieu rich in TGF_β_ and depleted of IL-23 favors the generation of non-pathogenic IL-10-producing Th17 cells while an environment with high levels of IL-23 support the expansion of autoreactive Th17 cells [11]–[14]. Th17 cells that support autoimmune disease are characterized by increased expression of cytokines including IL-33, surface markers including IL-23R and transcription factors including T-bet. Current results show that these pathogenicity-related genes are under-expressed by the Th17 cells generated by Sup ODN whereas the expression IL-10 is elevated (Fig. 4 and [12], [46]. In this context, the profile of Th17 cells generated by Sup ODN is similar to that induced by the immunosuppressive agent Tofacitinib, which also supports the generation of non-pathogenic Th17 cells [47].

A variety of inflammatory and autoimmune conditions are currently treated with anti-inflammatory drugs that down-modulate the immune system. Patients placed on immunosuppressive regimens are therefore at increased risk of disseminated fungal infection. An agent that suppresses inflammation while improving host resistance to extracellular pathogens would be of considerable therapeutic value. Previous studies established that Sup ODN could prevent and/or treat a variety of inflammatory and autoimmune diseases [2], [3], [5], [7], [9]. Current results suggest that Sup ODN also promote the generation of non-pathogenic Th17 cells, thereby enhancing host resistance to fungal infection. These findings support the further development of Sup ODN.

## Supporting Information

Figure S1 **Suppressive ODN do not alter Th1 or Th2 maturation under Th17 polarizing conditions.**
(TIF)

Figure S2 **Effect of suppressive ODN on STAT1 and STAT5 phosphorylation.**
(TIF)
