# Supplementary figures and images for: Suppressive Oligodeoxynucleotides Promote the Development of Th17 Cells
Source: PLoS One. 2013 Jul 2;8(7):e67991. doi: 10.1371/journal.pone.0067991 (PMC3699503; doi:10.1371/journal.pone.0067991)

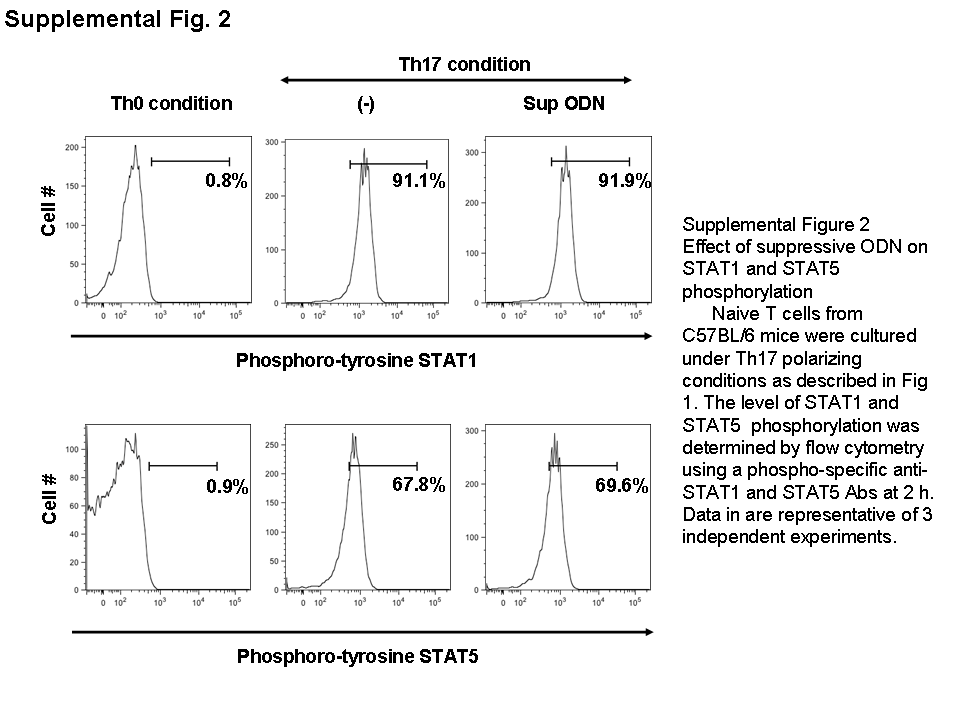

Supplement: Figure S2 — Effect of suppressive ODN on STAT1 and STAT5 phosphorylation. (TIF) [file pone.0067991.s002.tif]
